# Moving Toward the Next Generation of HMLS—Testing and Validating the Performances of Second-Generation SLAM Systems Compared to Predecessors

**DOI:** 10.3390/s25082488

**Published:** 2025-04-15

**Authors:** Lorenzo Teppati Losè, Fulvio Rinaudo, Nives Grasso, Cristina Bonfanti, Steffen Kappes

**Affiliations:** 1 Laboratory of Geomatics for Cultural Heritage (LabG4CH), Department of Architecture and Design (DAD), Politecnico di Torino, Viale Mattioli 39, 10125 Torino, Italy; fulvio.rinaudo@polito.it; 2 LabGeomatics, Department of Environment, Land and Infrastructure Engineering (DIATI), Politecnico di Torino, 10129 Torino, Italy; nives.grasso@polito.it; 3 FARO Technologies Italy S.r.l., Via G. Matteotti, 161/163A, 25086 Rezzato, Italy; cristina.bonfanti@faro.com; 4 Department of Technology & Innovation, FARO Europe GmbH, Lingwiesenstraße 11/2, 70825 Korntal-Münchingen, Germany; steffen.kappes@faro.com

**Keywords:** SLAM, TLS, accuracy evaluation, HMLS, point clouds

## Abstract

Among the different activities of the AEC (Architecture, Engineering, and Construction) sector, the documentation phase is pivotal and covers the entire lifecycle of a building or infrastructure. In the last decade, in the geomatic field, technology has evolved rapidly, and several instruments and techniques have become available to assist operators in this documentation process. Furthermore, the AEC sector is moving toward the extensive use of Digital Twins, and the research presented in this paper focuses on the technological solutions available today for creating the metric and geometric base of the Digital Twin at the service of AEC sector. Geomatics instruments and techniques are widely adopted in this framework, particularly HMLS (Handheld Mobile Laser Scanner). This research will evaluate the differences in performances between the first and second generation of HMLS based on SLAM (Simultaneous Localisation and Mapping) technologies in terms of accuracy, precision, level of detail, data density, noise, and other relevant characteristics. To address the research questions of this work, it was decided to perform a series of tests in an ad hoc test field following predefined acquisition strategies and procedures. A series of analyses were then conducted on the processed data to evaluate several factors, particularly georeferencing of HMLS data, features analyses on specific areas, Cloud-to-Cloud analysis, and cross-sections analysis.

## 1. Introduction

The documentation phase is a pivotal activity in the AEC (Architecture, Engineering, and Construction) sector, and it is applied in the entire lifecycle of a building or infrastructure (from its design, via its construction, through its maintenance life until its demolition). The documentation activities are thus encapsulated in all the different phases of the Building Life Cycle (BLC). The BLC is a well-established approach and can be applied to several aspects of a building’s life [1,2,3].

In the last decade, in the geomatic field, technology has evolved rapidly, and several instruments and techniques have become available to assist operators in the documentation process of the AEC sector. This sector is moving toward the extensive use of the Digital Twin. The concept of Digital Twin is complex and can be applied in several industries and contexts; in the AEC sector, it has a long history of development [4,5,6,7].

Digital Twins can support intelligent green buildings [8], assist the optimisation of energetic performances and thermal comfort [9,10], provide a valuable contribution to the building maintenance [11], support decarbonizing operating buildings [12], assist decision-makers in the management of complex buildings like hospitals [13] or enhance fire safety management operations [14].

It also needs to be reported that the concept of Digital Twin is also moving from the building scale to the urban one, with all the connected challenges, as the experiences described in [15,16,17,18].

Nevertheless, the geomatic contribution to the definition of the metric components of the building is crucial [19,20,21]. In the market, there are different approaches and solutions for the documentation of these assets and the creation of the metric context of a digital twin, as well as different levels of information, details, and accuracy that can be achieved depending on the selected approach.

This paper focuses on the technological solutions available today for creating the metric base of the Digital Twin used to implement all the other information and features of this approach.

The range of these technological solutions is vast and constantly evolving, and the market is following the trends of the AEC industry to answer its needs. The first level of documentation is nowadays provided by the use of 360° cameras and web-based platforms such as Cupix [22], Holobuilder [23], and Matterport [24]. These solutions can be seen as the evolution of traditional virtual tours where additional information is added to the 360° images, serving as the base for data questioning and management. The metric components are managed differently from the various solutions available on the market; however, these platforms are generally conceived as high in content information and low in accuracy (in a range of a few centimetres). The strengths of these solutions belong to the rapidity and easiness of use (simple and fast training required for the operators), the possibility of implementing multitemporal monitoring of buildings and construction sites at a relatively low cost and, recently, the possibility of integrating them with 3D models: point clouds and BIM (Building Information Modelling). Another important use of these systems exists in the documentation process’s design phase, where they can be adopted to plan the field activities in detail [25].

At the current stage of development, these systems can be seen as a preliminary tool in the creation of the metric base for the Digital Twin; to reach the level of detail and accuracy that is often needed for application in the AEC sector, it is necessary to move toward the use of other technologies.

It must be underlined that these kinds of cloud-based platforms are evolving for archiving, managing, and integrating data with multitemporal approaches and sharing images, plans, alphanumerical information, metric point clouds, and CAD models. All information is converging in a single project, working as a unique centre for data management and with a work area available online for all the different stakeholders, with the possibility of also implementing various levels of access (it is, for example, the case of Holobuilder, now becoming FARO Sphere XG^®^).

In this framework, geomatic instruments and techniques are widely adopted to generate 3D metric products that will be the metric base of the Digital Twin. In the case of the AEC sector [26], it is possible to recall the deployment of techniques based mainly on LiDAR (Light Detection And Ranging) [27] and automatic digital photogrammetry, mainly from UAS (Uncrewed Aerial Systems) [28].

Among the LiDAR technology, it is necessary to recall the rapid evolution of MMS (Mobile Mapping System) and, in particular, of HMLS (Handheld Mobile Laser Scanner) that invested several sectors in recent years and also AEC. In particular, solutions based on SLAM (Simultaneous Localization And Mapping) algorithms are now widely available and adopted for the documentation process [29,30].

Moreover, one of the focuses of this research is to evaluate the differences, if any, between the first (2019) and second generation (2022/2023) of HMLS based on SLAM technologies in terms of accuracy, precision, level of detail, density of data, noise, and other relevant characteristics when documenting the Built Heritage.

## 2. Materials and Methods

To address the research questions of this work, it was decided to perform a series of tests in an ad hoc test field following predefined acquisition strategies and procedures.

**Figure 1:**
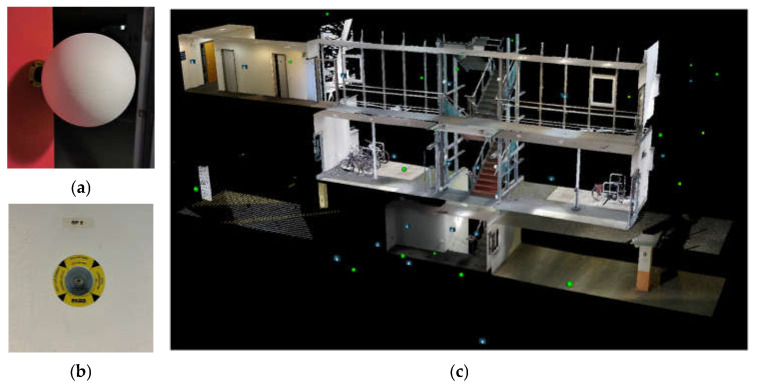
Example of survey grade sphere placed on the test track (**a**). Metal boss of one of the GCPs (**b**). Axonometric view of the distribution of part of GCPs (**c**).

The test track for performing the acquisition and analyses presented in this article was set up at the FARO offices in Stuttgart. For the aim of this research, the building represented an optimal solution for different reasons. First, it was provided with a fixed network of GCPs (Ground Control Points): several metal bosses are fixed in the building walls and measured via a traditional topographic approach using a total station. Moreover, the points are distributed all over the building on different floors, from the underground garage to the second floor above ground. Polystyrene survey-grade spheres of various dimensions can be placed on the bosses and used for data processing (Figure 1).

Furthermore, the possibility of acquiring data on broad areas starting from the outdoors and moving inside different typologies of indoor spaces located on various floors represents a perfect test field for the HMLS systems.

Finally, the building is characterised by different materials that allow the testing of the system’s responses to challenging textures and surfaces (e.g., wide area glass walls, stairs and corridors, indoor and outdoor areas present along the trajectory).

### 2.1. Instruments Description and Specifications

The acquisitions at the test track involved a series of different instruments and techniques. For this research, three instruments will be considered and analysed: one TLS (Terrestrial Laser Scanner) will be used as reference data, and three different HMLS systems will be evaluated.

**Figure 2:**
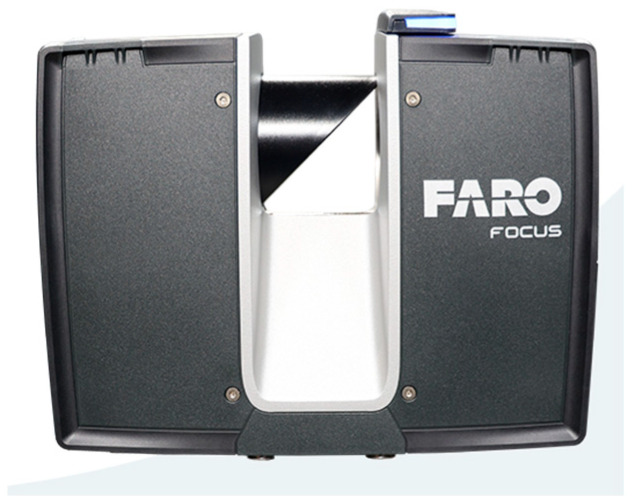
FARO Focus Premium.

The FARO (FARO Europe GmbH, Korntal-Muenchingen, Germany) Focus Premium is the TLS system used to acquire the reference dataset. The instrument is shown in Figure 2, and the main specifications are reported in Table 1.

The parameters for the acquisition are the following: resolution ¼, quality 3× with a mean number of 43 million points for each scan and a mean step between points of 6.1 mm at 10 m from the sensor (acquisition time 02:30 min + 30 s in colour acquisition with Panocam Ricoh (Tokyo, Japan) Theta Z1).

The three HMLS systems tested in this research are

**Figure 3:**
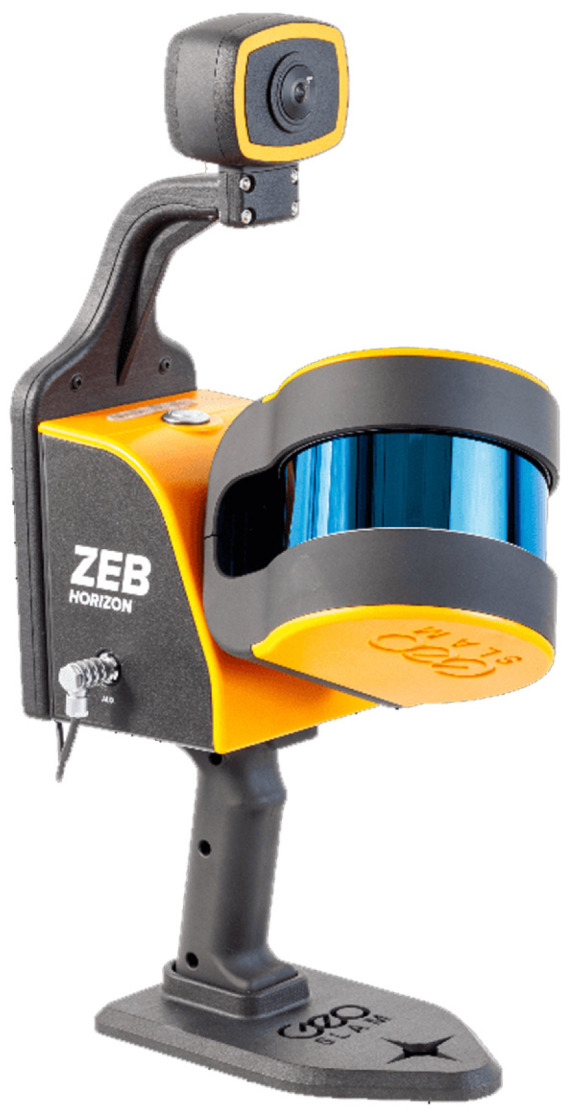
GeoSLAM Zeb Horizon.

**Figure 4:**
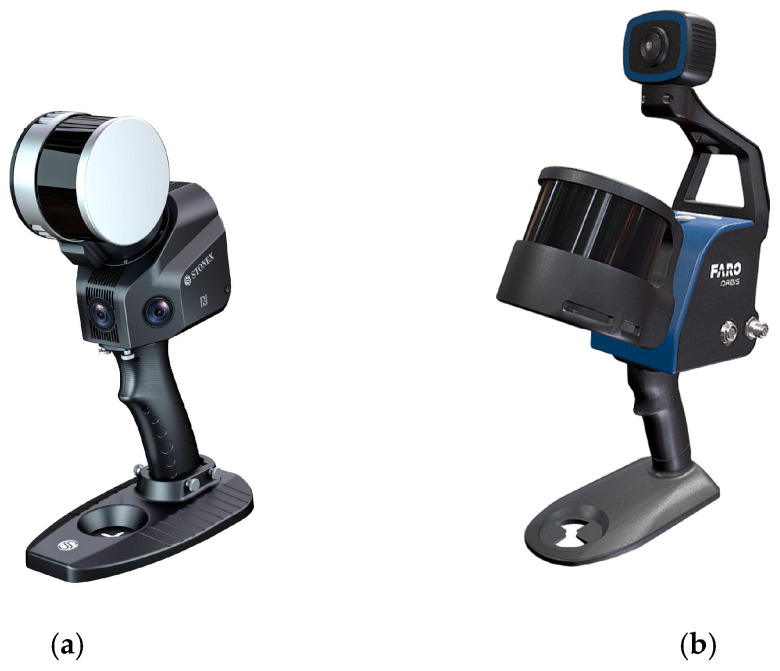
Stonex X120 GO (**a**) FARO Orbis (**b**).

- GeoSLAM (FARO Europe GmbH, Korntal-Muenchingen, Germany) Horizon (Figure 3 and Table 2) as the first generation of systems;
- Stonex (Monza, Italy) X120 and the FARO (FARO Europe GmbH, Korntal-Muenchingen, Germany) Orbis as the second generation of devices (Figure 4 and Table 3).

### 2.2. Acquisition Strategies

**Figure 5:**
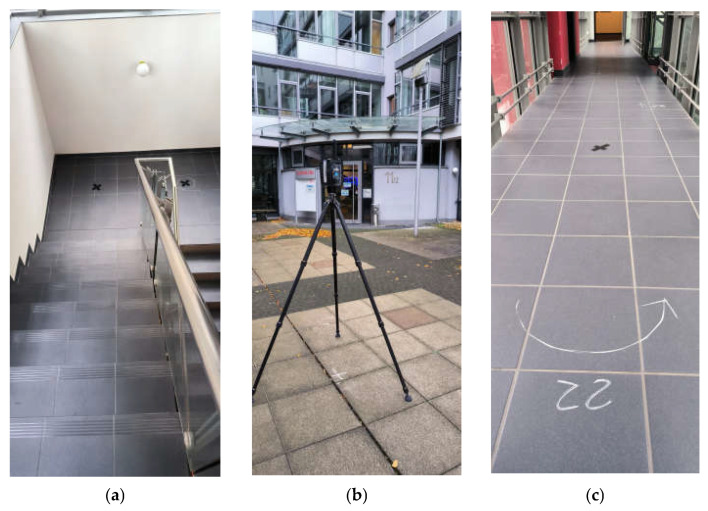
Acquisition setup for the TLS reference dataset and the different HMLS systems. Static acquisition positions (**a**), acquisition with the TLS (**b**), turn of the HMLS path marked on the ground (**c**).

The acquisitions were carefully planned to ensure a fair comparison between the different instruments, and the test track was set accordingly. The first step was to acquire all the areas of the test track that would have been used for the test with the HMLS and TLS. Polystyrene calibrated spheres were placed all over the GCPs on the metal bosses, and the scan positions of the static laser were numbered and marked on the ground with chalk (Figure 5a–c).

**Figure 6:**
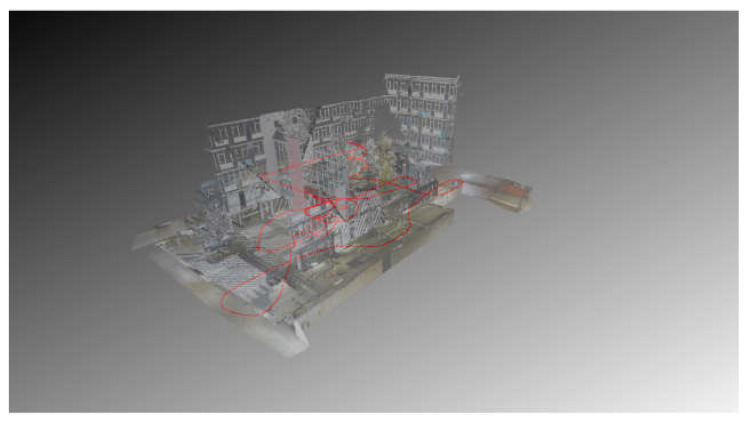

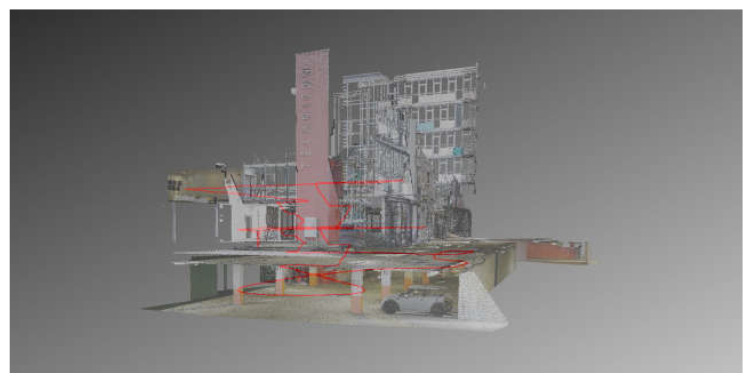
Acquisition path followed by the HMLS (in red) overlayed on the TLS point cloud.

The strategies followed for the HMLS acquisition were similar: the closed path to follow during the acquisitions was tested beforehand and marked on the ground with chalk. In particular, it was necessary to mark the turns and direction changes for the operator performing the acquisition (Figure 5c). An overview of the path followed by the operator is shown in Figure 6. The time needed to complete the acquisition with the HMLS was around 15 min.

## 3. Data Processing

This paragraph will briefly describe the data processing. Consolidated approaches and dedicated proprietary software were used for each considered system. Several options are available during the HMLS data processing, and often, it is not easy to relate the chosen approach with the results achieved for each instrument. To make the comparison of the solutions as fair as possible in this preliminary set of tests, it was decided to follow the standard pipeline as indicated in the instrument manual and avoid applying multiple filters during the processing. This additional topic was temporarily not investigated further but will be analysed and discussed in future research work. It should also be noted that the parameters that the user can customise in the processing of the different HMLS data may vary significantly in the different software solutions. Referring to the latest software available at the time of the data processing, the parameters that the user can modify, and thus the type and quality of data exported at the end of the processing, are quite different for the three considered HMLS systems; more details are available in Section 3.1.2 for the Stonex X120GO and 3.1.3 for the GeoSLAM Horizon and FARO Orbis.

The final aim of the processing was to obtain the most “raw” dataset to avoid adding other non-controllable variables. For this reason, in this first research, topographically measured GCPs (Ground Control Points) were not foreseen for the HMLS data processing, as the different software deals differently in managing this information. Nevertheless, the datasets have been processed by basic SLAM algorithms without applying rigid or non-rigid transformation on control points. This leads to the need to georeference the HMLS data with different strategies, as is reported and described in Section 3.2. The use of GCPs for HMLS data processing is a topic worth researching and needs dedicated tests and evaluations. Nevertheless, this research aimed to evaluate the performances of two different generations of HMLS for the metric and geometric definition of built heritage.

### 3.1. Data Processing for Each Instrument

#### 3.1.1. TLS Data Processing

The TLS dataset was processed using the FARO SCENE 2024 software and following the new pipeline for scans’ registration. The traditional processing workflow foresaw two traditional main steps: (i) cloud-to-cloud registration and (ii) target-based registration, but since the 2023 release, the software has permitted the scan registration using the Interactive Registration process.

**Figure 7:**
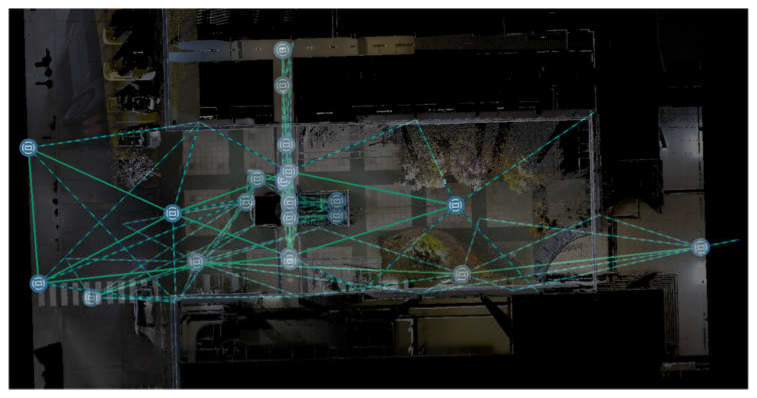
TLS data Plan view and hybrid connections between scan positions (continuous lines) and target scans positions/referencing (dashed lines) resulting from the bundle optimisation.

**Figure 8:**
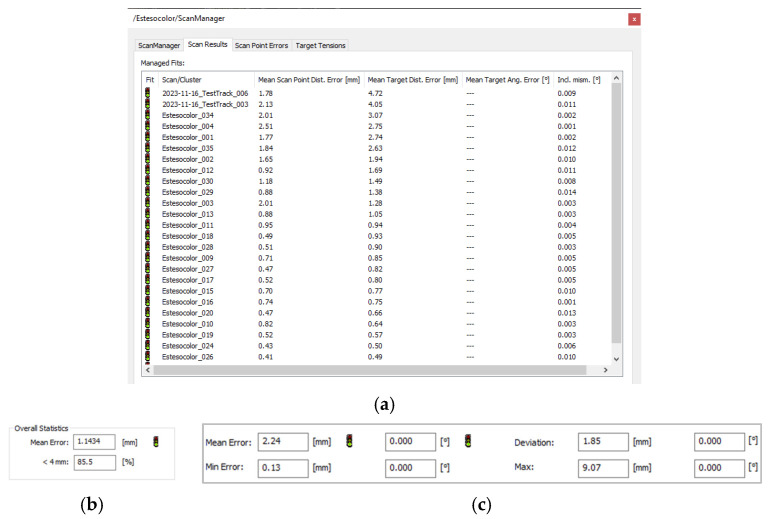
Results from hybrid registration process (**a**) with C2C (**b**) and targets based for registration and referencing (**c**) overall statistics.

In our case, the new hybrid bundle optimisation workflow has been used to align and reference scans on the C2C (Cloud to Cloud) approach together with the targets-based one (Figure 7), improving the results and reducing the computation time (Figure 8a–c).

#### 3.1.2. Stonex X120GO

At the time of writing, the software provided by Stonex offers a few customisable parameters to optimise and reprocess the acquired data. One key feature is the ability to recompute the acquisition trajectory using IMU/LiDAR data, enabling more accurate mapping of recorded assets. Additional processing options include the application of filters such as pedestrian removal, de-noising, and thinning. The point cloud is coloured using RGB camera data, followed by a general optimisation that removes outliers and cleans double surfaces. An optional step involves processing the camera data, which includes stitching and creating panoramic images.

For the Stonex X120GO, as for all the other HMLS systems, the processing was maintained as simple as possible using the dedicated proprietary software GoPost (v 2.0). The One-Click solution was adopted, and the only parameter considered was the stability setting. This parameter deals with the features of the scene (more or less complex) and needs to be set according to them. After some tests for the specific case study considered, it was decided to use the stability parameter 3, which delivered the best results. During the different tests, and in the analysis later completed, it has been noted that the GoPost software automatically applies some filtering and noise reduction on the collected data. In the software version available at the time of processing, it was not possible to customise this parameter and control this part of the processing. This aspect was thus considered when analysing the data in comparison to the other HMLS systems. The final processed point cloud of the X120GO is composed of around 22 million points.

#### 3.1.3. GeoSLAM Horizon and FARO Orbis

FARO Connect (v 2023.1) is the software package for processing and viewing point clouds and 360° image data and for creating cleaned, thinned, coloured, georeferenced point clouds primarily captured from the FARO Orbis and ZEB systems. Powered by GeoSLAM’s proprietary SLAM algorithm, Connect is the all-in-one software for the local processing of mobile data, connected to the optional FARO SphereXG^®^ cloud-based platform for viewing, sharing, and collaborating. The software allows the reprocessing of field-acquired data (in the case of Orbis, also via Stream App) using different default or advanced algorithms, where parameters such as automated filtering (including outlier, noise, thinning and transient workflows), colouring, acquisition contexts, georeferencing, etc., can be used mainly as automated or customisable workflows, based on the specific application. In addition to these options and the “traditional” SLAM processing integrating LiDAR and IMU data, different mobile scans can be aligned and georeferenced using rigid and non-rigid transformations within a single project.

**Figure 9:**
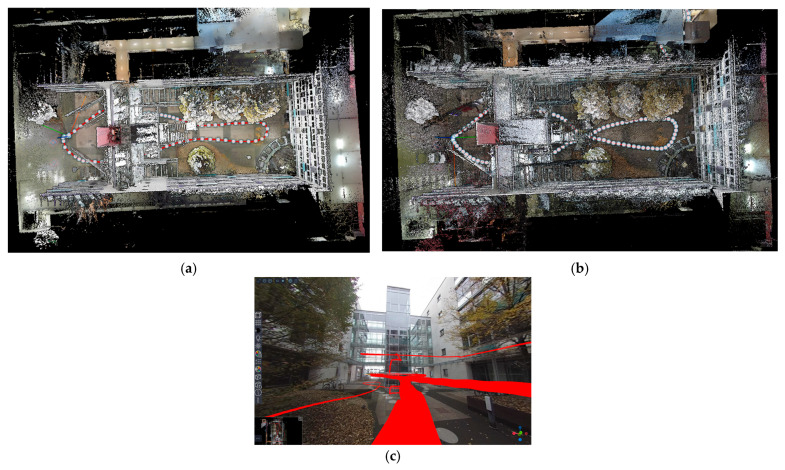
Processed GeoSLAM Horizon dataset (**a**) and FARO Orbis one (**b**) integrated with trajectory and Vision images. A 360° image opened in Connect (**c**).

As previously motivated, also in this case, default processing was applied to both datasets, which included colouring and basic point cloud filtering (Figure 9). A specific option is dedicated to Orbis data to extract Flash scans acquired along the path. In the FARO Connect software, several options are available for the operator to fully customise the processing and apply different levels of filtering, enabling the possibility of exporting both “raw” point clouds and the results of the filtering algorithms applied with different coefficients. The final processed point cloud of the Horizon is composed of around 100 million points, and the Orbis one has around 200 million points.

### 3.2. Georeferencing Strategy and Results

The georeferencing strategy was one of the first problems addressed after individual data processing for each device. There is, of course, the possibility of implementing specific approaches in the different processing software of the HMLS systems employed; however, for the reasons reported in the previous section, it was decided to follow a simple strategy directly applied to the processed point clouds of the different HMLS. This approach was also chosen to ensure a fair comparison between the other datasets, with the georeferencing strategy of each solution being different and considering that the use of GCPs in the processing phase may add additional constraints that need to be independently evaluated.

FARO Focus Premium has been used as a reference to solve this issue and georeferencing the datasets in the same reference system. As previously described, this dataset was processed following consolidated strategies, thanks to the permanent GCPs network of the test track.

The HMLS datasets were then co-registered with the TLS data using an n-point registration. In this approach, the TLS point cloud is used as a reference, and its position is fixed while the different HMLS datasets are moved to be co-registered with the TLS one. In any of these cases, the point clouds are considered already scaled; thus, this parameter is not influenced by this approach.

For completing the georeferencing, a set of 15 corresponding points has been created and used for all the HMLS point clouds. These 15 points were chosen to be identifiable on the different datasets and were all used in the process; moreover, they were distributed all over the building from the garage up to the 1st floor (the area covered by the LiDAR acquisitions).

**Figure 10:**
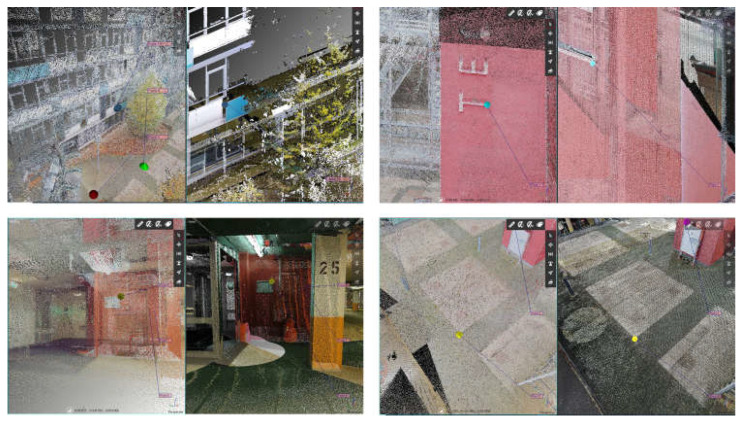
Example of common points chosen as a reference for the N-point alignment.

**Figure 11:**
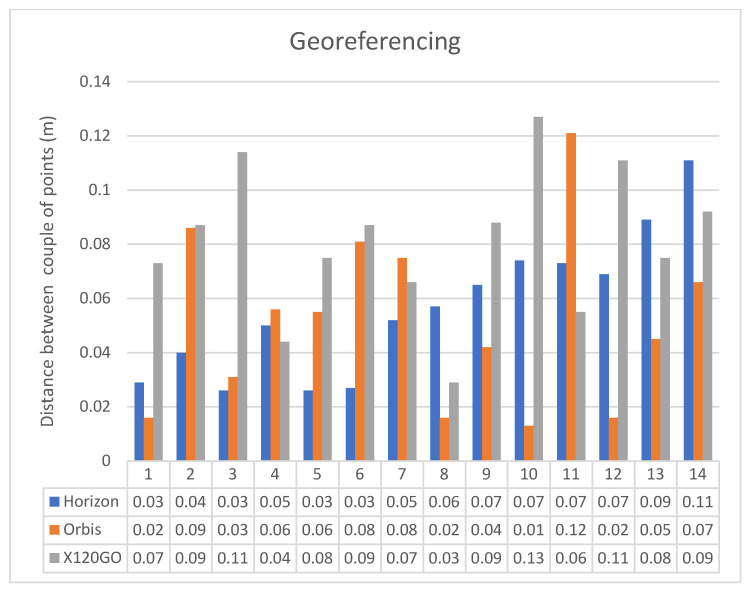
Graphical representation of the distances between couples of points in the n-point registration.

Some examples of the points chosen are shown in Figure 10, while the results of this operation are reported in Table 4. A graphical representation of distances between points is reported in Figure 11.

It is essential to report that with this approach, it is also possible to achieve the first analysis of the quality of the HMLS point clouds. Analysing the deviation between the position of the points used, it is possible to have a first idea of the overall quality of the point cloud and then start to highlight if there are any evident drift errors, both locally and globally. Of course, human error in picking the points should be considered. For this reason, the co-registration process has been supervised by multiple operators and repeated numerous times to confirm the results.

Starting from the distance between the points used as a reference in this process, it is possible to highlight some considerations. While the performances of the GeoSLAM Horizon and the FARO Orbis are comparable, the ones of the Stonex X120 GO are slightly worse. The mean distance between the points of the first two instruments is around 5 cm, while the latter is 8 cm. For this first research stage, it was decided not to perform an ICP registration to finetune the results. This choice is mainly related to the typology of the HMLS point clouds, which are noisier than the TLS ones. To successfully apply an ICP registration, it would have been necessary to reduce the noise and subsample the data to be more homogenous. It has to be considered that several parameters are involved in processing each dataset and that they differ among the different software solutions. Using an n-point registration approach seemed more reasonable in comparing the various systems fairly. Nevertheless, implementing this approach allowed for the georeferencing of all the data in the same reference system and, thus, proceeded with a more detailed analysis of the point cloud quality.

## 4. Data Analyses

The analysis of the HMLS data was conducted on selected sample areas to evaluate the different instruments’ performance in defining the building’s geometric features. Moreover, the regions selected for the various analyses were chosen on the three floors interested by the acquisitions (underground, ground, and first floor) and represent different features and materials (walls, doors and windows, plaster, metal, glass, etc.).

Three types of analyses were performed:

- density and features analyses,
- Cloud2Cloud analyses,
- cross-section analyses.

### 4.1. Density and Features Analyses

#### 4.1.1. Density Analyses

**Figure 12:**
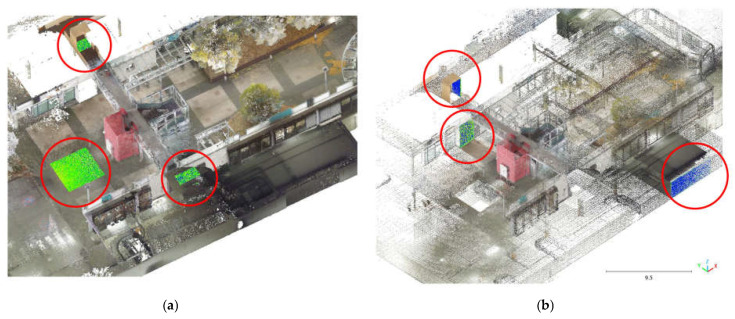
Position of the sample area (red circles) for density and features analysis. (**a**) Horizontal regions. (**b**) Vertical regions.

The density and features analyses were performed using the open-source software CloudCompare (v 2.12.3) [31]. For this specific analysis, six sample areas were identified: three with a horizontal development and three with a vertical development. The horizontal and vertical regions were distributed over the three floors considered in the acquisition phase. The positions of the sample areas are reported in Figure 12.

**Figure 13:**
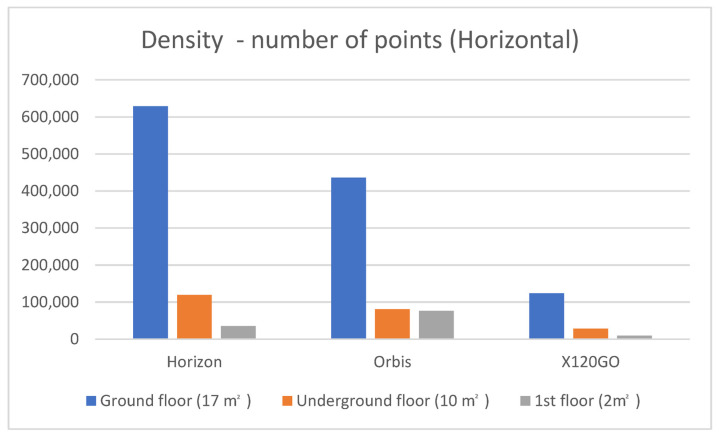
Graphical representation of the horizontal density analyses on the three selected sample areas.

The density analyses on the horizontal surfaces considered three types of floorcoverings on the three floors: concrete for the underground, self-locking tiles on the ground floor and moquette on the first floor. The ground floor is the biggest in terms of square metres (17 m^2^), followed by the underground (10 m^2^) and a small area on the first floor (2 m^2^). The results of the analysis are reported in Table 5 and Figure 13.

For the horizontal density analyses, it is interesting to report some considerations. For the ground floor, the sensor providing the higher number of points is the Zeb Horizon. On the underground floor, Zeb Horizon and FARO Orbis are delivering similar results with a slightly higher density of the Zeb Horizon. On the first floor, the FARO Orbis is the system with the higher density, double that of the Zeb Horizon. In all three scenarios, the X120GO has the lower density, which we somehow expected considering the sensor’s specifications and the filtering performed by the GoPost software during the processing.

**Figure 14:**
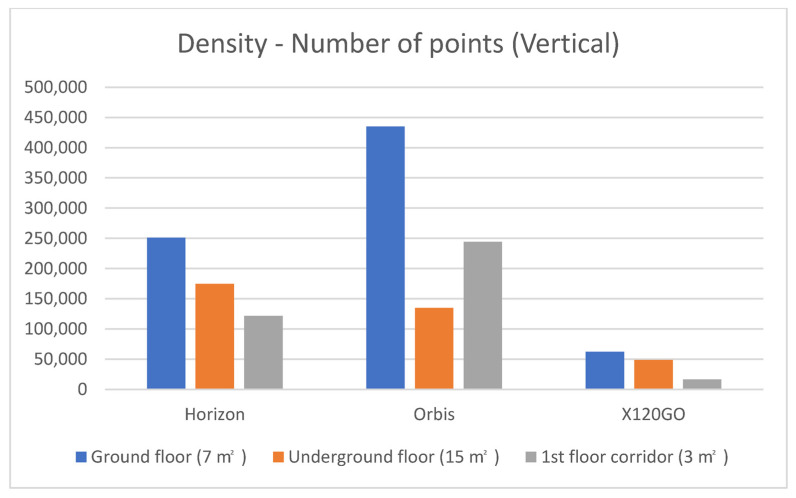
Graphical representation of the vertical density analyses on the three selected sample areas.

The vertical density analysis was achieved following the same procedure as the horizontal one. Among the three areas selected, the underground is the biggest one in terms of square metres (15 m^2^), followed by the ground floor (7 m^2^) and finally, the first floor (3 m^2^). Materials and textures differ in the three areas: painted plaster for the underground and first floor and metal and glass for the ground floor. Results of the density analysis on the selected vertical surfaces are reported in Table 6 and Figure 14.

For the vertical density analyses, results are slightly different compared to the horizontal one, with a better performance regarding the absolute number of points of the Faro Orbis.

On the ground floor, the system with the highest number of points is the FARO Orbis (almost double of the Zeb Horizon). On the underground floor, the Horizon has a slightly higher number of points but is nearly identical to the Orbis. For the first floor, the Orbis is again the sensor generating the higher number of points.

#### 4.1.2. Feature Analysis

The second analysis performed on the selected areas is the feature analysis carried out again with the software CloudCompare. Specifically, two analyses were completed: number of neighbours and a roughness analysis. These additional evaluations were performed to deepen the results of the density analysis. The absolute number of points in a specific area cannot be considered an indicator of the quality of the data alone and needs to be integrated with other indexes. The tool used in CloudCompare is the “compute geometric features” that requires the selection of some parameters by the operator. The primary consideration concerns the radius of the spherical region that will be used to perform the computation (it is also defined as kernel and is the radius of the sphere centred on each point). For this research, a radius of 0.02 m was chosen, a value generally conventionally accepted as the precision for an architectural survey.

Estimating the local density via the number of neighbours allows the addition of more information for evaluating the instrument’s performances, indicating the distribution of the points in the considered sample areas.

**Figure 15:**
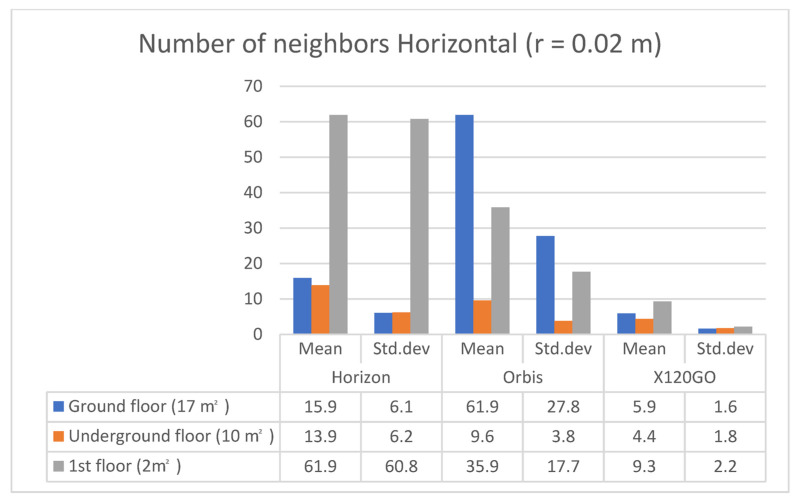
Graphical representation of feature analysis. Number of neighbours on **horizontal** surfaces.

The analysis was performed for all three horizontal sample areas, and results are reported in Table 7 and Figure 15.

**Figure 16:**
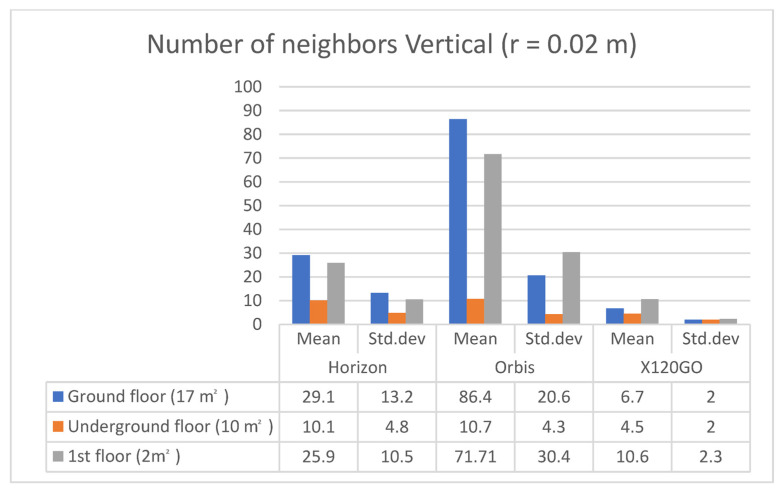
Graphical representation of feature analysis. Number of neighbours on **vertical** surfaces.

For the ground floor, the Orbis has a higher number of neighbours, followed by the Horizon; for both, the standard deviation is almost half of the mean. For the underground floor, the Horizon is performing slightly better in terms of the mean of the number of neighbours, and again, the standard deviation for both Horizon and Orbis is close to half of the mean. Finally, for the first floor, the Horizon has a higher number of neighbours; however, in this case, the standard deviation is equal to the mean for this instrument. The Orbis follows the trend of the other two areas with a standard deviation equal to half of the mean. In general, it is interesting to note that the X120GO, despite having a lower number of points, is also achieving lower standard deviation values than the other two sensors; here again, we need to consider the filtering of data applied during the processing. The same analysis was repeated using the same parameters for the three vertical surfaces, and the results are reported in Table 8 and Figure 16.

The vertical analysis presents results similar to those of the horizontal surfaces. In this case, the Orbis is generally the sensor with more neighbours and a standard deviation around half the mean. In this scenario, the X120GO seems to be the sensor with a lower number of neighbours but with a contained standard deviation.

The second feature analysis performed was the roughness analysis. This index comprises a series of values that indicate the distance between each point considered and the best-fitting plane computed on the nearest neighbours. Also, for this analysis, the radius of the spherical region around each point was set to 0.02 m in performing the computation.

**Figure 17:**
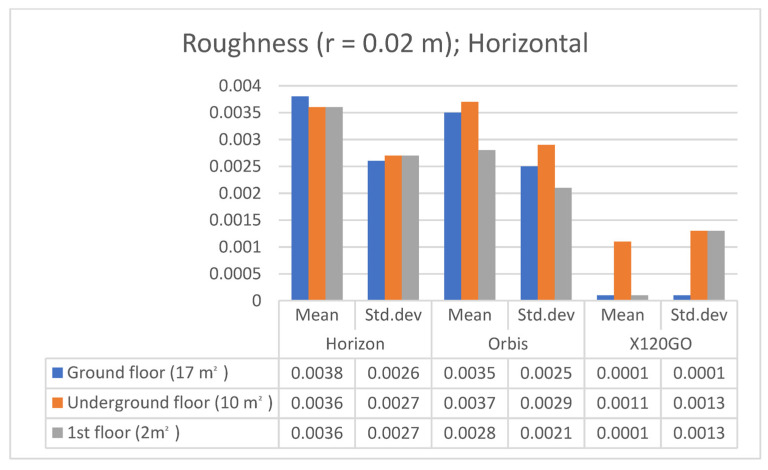
Graphical representation of roughness on **horizontal** surfaces.

The results of this analysis are reported in Table 9 and Figure 17.

Analysing the roughness of the horizontal surfaces allowed us to identify specific trends. The X120GO seems to be the sensor achieving the lowest level of roughness. However, the standard deviation associated with it is exceptionally high (especially for the underground and first floor). The other two HMLS present a higher roughness, but the standard deviation in these cases is almost equal to the mean values.

**Figure 18:**
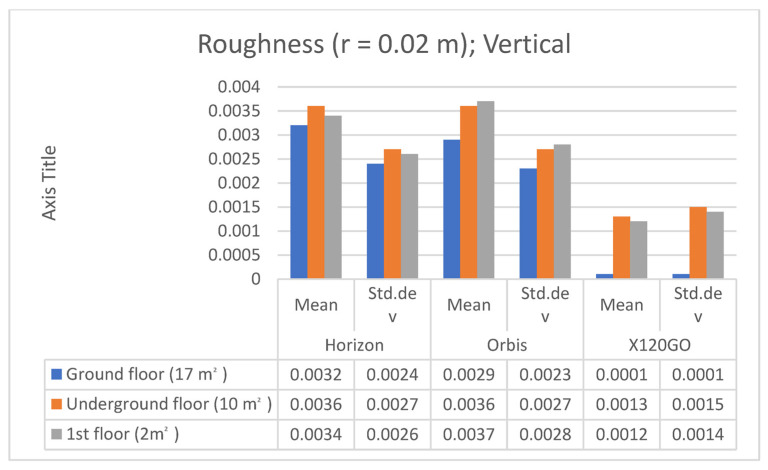
Graphical representation of roughness on **vertical** surfaces.

The same analysis was achieved on the vertical surfaces of the three sample areas, and the results are reported in Table 10 and Figure 18.

The results of the roughness analysis for the vertical surfaces mainly confirm the results achieved on the horizontal surfaces. Horizon and Orbis are performing in the same way, both in terms of mean and standard deviation. X120GO is again the one with the lowest roughness values, but in this case, the standard deviation is comparable with the mean for the other two systems.

### 4.2. Cloud2Cloud Distance Analyses

**Figure 19:**
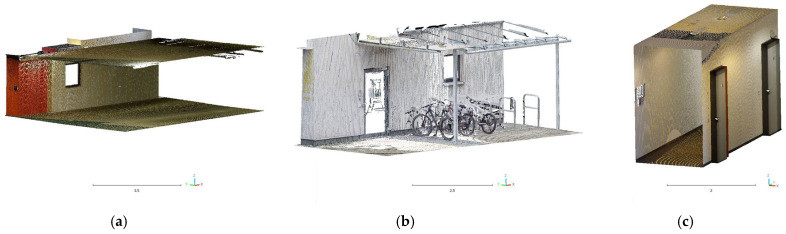
Sample areas of the C2C analysis. (**a**) Area 1—Underground. (**b**) Area 2—Ground Floor. (**c**) Area 3—First Floor.

To evaluate the overall performances of the HMLS systems, another analysis was implemented in the other three areas of the test site: the Cloud2Cloud distance analysis. This tool is present in different software for point cloud analysis and management, and for this research, it was decided to use the open-source solution CloudCompare again. The Cloud2Cloud distance computes the distances between two point clouds: one selected as reference and the other as compared. The reference cloud is derived from the TLS acquisition (Section 2.2) that was considered ground reference using a strategy well documented in the literature. The three areas selected are again on the three different floors of the building acquired with the HMLS (Figure 19). The maximum distance for this analysis was set at 0.1 m to exclude possible outliers. The analysis was repeated with the same parameters for all the three HMLS considered.

**Figure 20:**
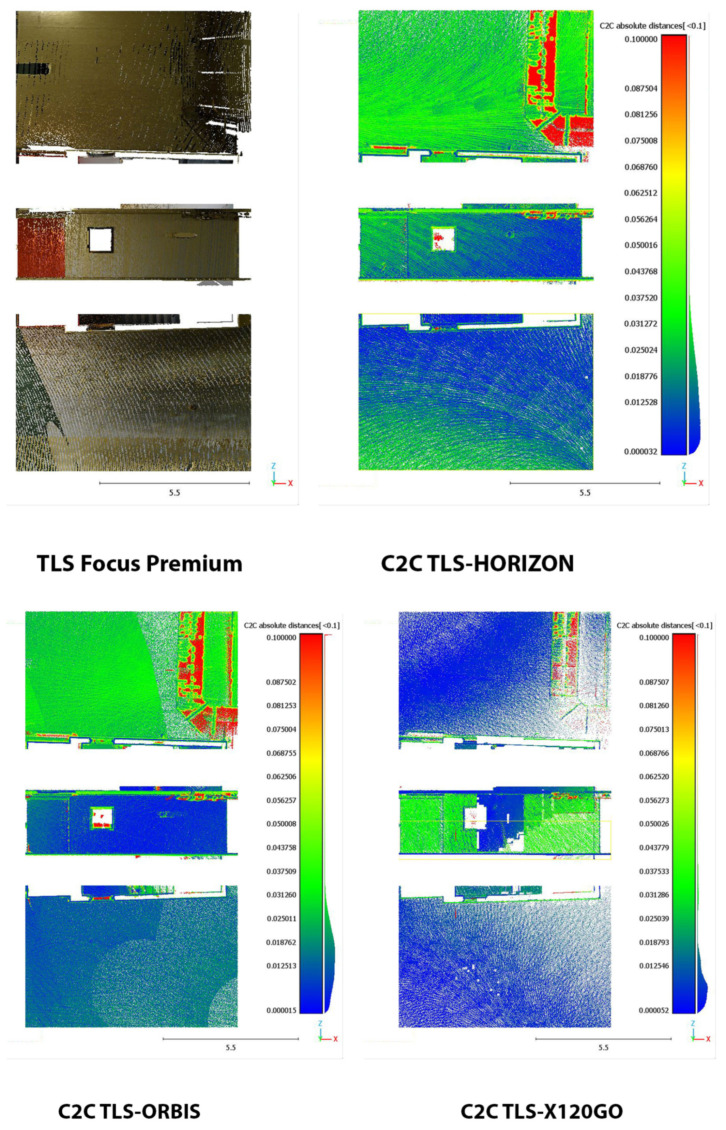
C2C analysis Area 1 underground. False colour representation.

The area selected for the C2C analysis on the underground floor contains a portion of the garage with a window on the stairs leading to the ground floor (Area 1). The results of the analysis are reported in Figure 20 and in Table 11.

In terms of absolute values, the Orbis and the X120GO are the systems performing slightly better. However, if we look at the spatial distribution of the error shown in Figure 20, it is possible to notice a different behaviour of the data. In the Horizon dataset, the higher deviations are distributed on the ceiling and the floor; in the Orbis, they are mainly distributed on the ceiling, while in the X120GO, on the vertical wall. All three systems present higher errors on the metal pipe for the air system management; this issue is probably related to the reflective material of the pipe.

**Figure 21:**
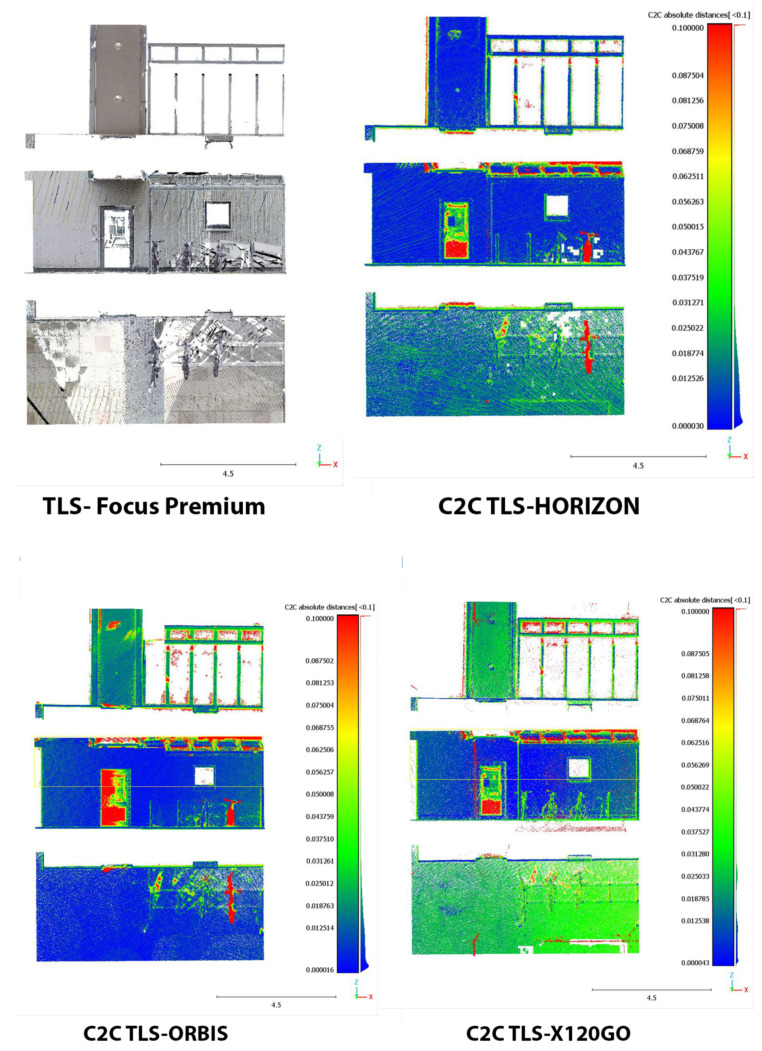
C2C Analysis for Area 2 on ground floor. False colour representation.

The second area is located on the ground floor and is one of the building’s entrances with a glass door and a glass shed for bicycle parking (Area 2). The results of the C2C analysis on this area are reported in Figure 21 and in Table 12.

In terms of absolute value, the Horizon and Orbis are performing in a similar way while the X120GO is less performing in this area of the building with only 44% of the point with a distance with the reference TLS below 2 cm.

From the point of view of error distribution, in this case, the Horizon presents the highest deviation on the floor, the Orbis on the ceiling, and the X120GO on both.

**Figure 22:**
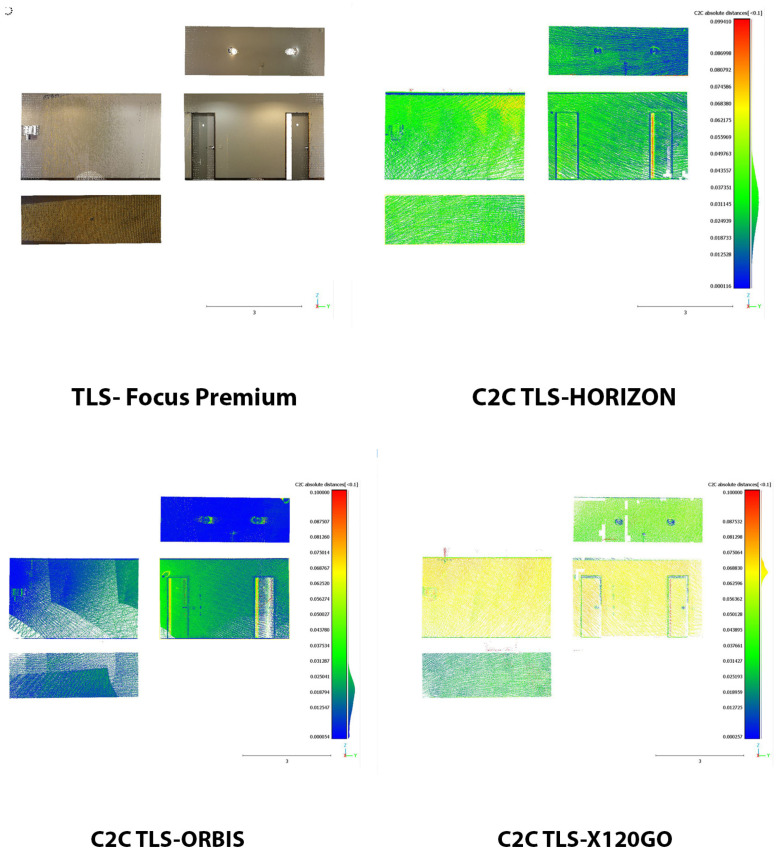
C2C Analysis for Area 3 on the first floor. False colour representation.

The last area was selected on the first floor in one of the office corridors (Area 3). The results of this analysis are reported in Figure 22 and in Table 13.

The scenario and the performances of the HMLS are different in this area. Overall, both the Horizon and the X120GO faced a drastic drop in the percentage of points with a distance under 0.02 m from the reference point cloud. For the X120GO, we have to reach the 0.08 m to comprehend the majority of points in the selected range. On the other hand, the Orbis is performing as well as in the other two areas with almost 80% of the points with a distance from the reference cloud of less than 0.02 cm. Looking at the spatial distribution of error, it is possible to notice that, for the Horizon and the X120GO, the deviations are distributed all over the different surfaces, while for the Orbis, the higher errors are localised on a small portion of the ambient.-

### 4.3. Analyses on Cross-Sections

**Figure 23:**
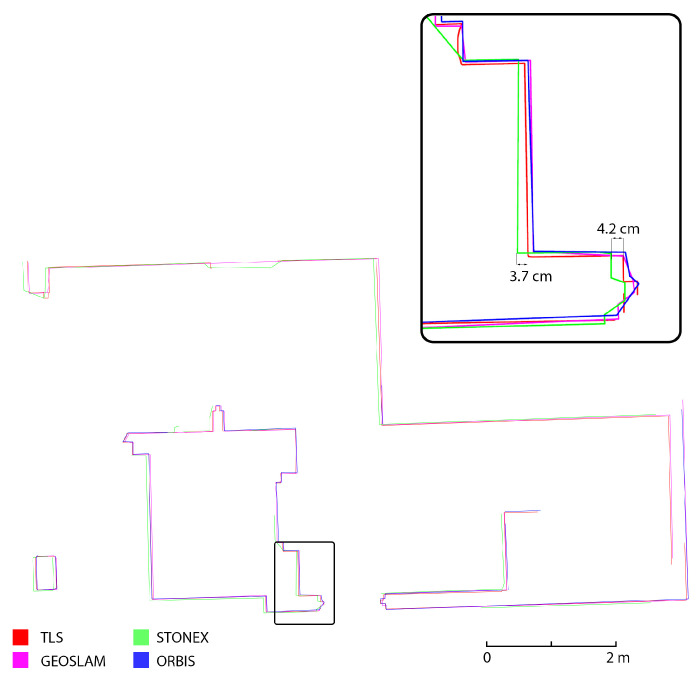
Horizontal section of a portion of the underground floor (Area 1).

**Figure 24:**
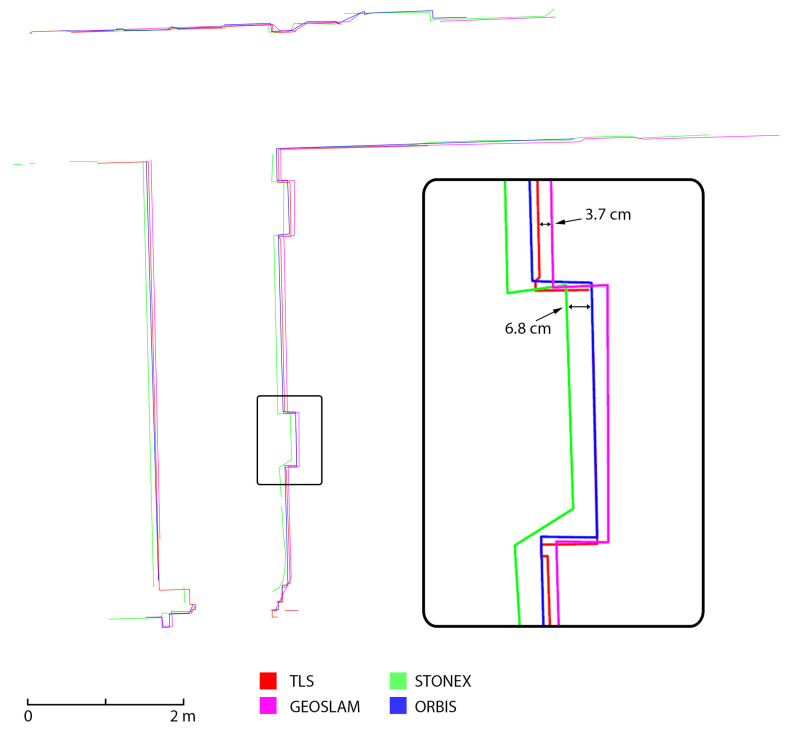
Horizontal section of a portion of the first floor (Area 2).

**Figure 25:**
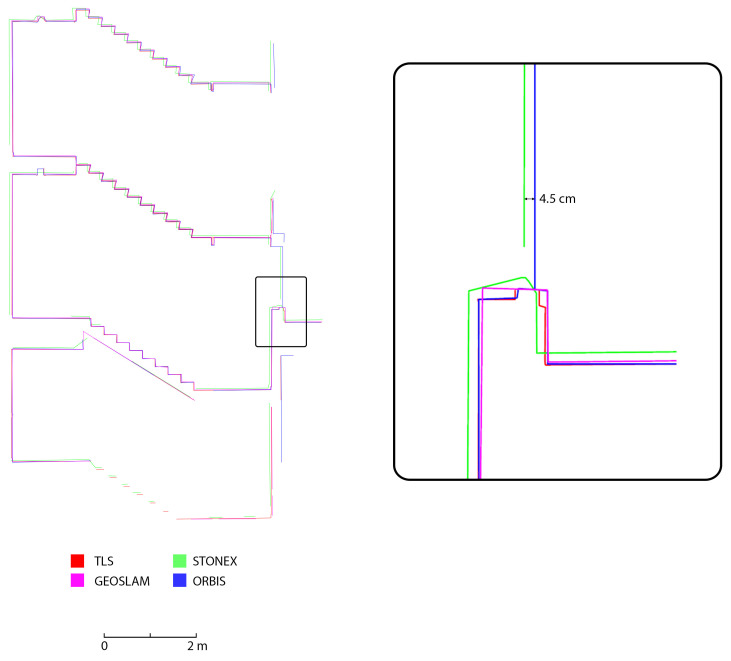
Vertical section of a portion of the stairs (Area 3).

The last analysis performed was achieved on the semiautomatic generation of cross-section profiles. This analysis was intended as a means to evaluate the ability of the different HMLS systems to represent the considered building, especially when the final aim of the survey is the generation of traditional 2D drawings. Three different section profiles were generated: two horizontal sections (underground and first floor) and a vertical cross-section on the stairs that connect the underground, ground, and first floor. The sections were created using the software PointCab Origins [32], which allows the setup of the section planes, their thickness and position and the generation of a 2D raster image directly from the point clouds. The sections (Figure 23, Figure 24 and Figure 25) were generated for all three HMLS datasets and for the TLS one that was used as a ground reference, also in this analysis. It is clear that those sections are not intended as architectural drawings but as the materials used as a basis for their creation.

The results achieved via this analysis confirm what already emerged from the results previously presented. In all three cross-sections considered, the FARO Orbis is the system with the best performance in terms of adherence to the TLS. This is verified both in terms of the completeness of the section profile and in terms of deviation between the reference dataset and the Orbis. It needs to be reported that the section profiles are often completely overlayed. The second best-performing device is the GeoSLAM Horizon, which, however, presents a deviation of a few centimetres in comparison to the TLS (2 to 4 cm), especially in the horizontal section. Finally, the Stonex X120GO has a higher deviation also in the vertical section, together with the horizontal one (from 3 to 7 cm).

## 5. Discussion

The test performed on the considered HMLS systems allowed us to highlight some considerations in the evolution of these systems over time. Despite being a preliminary analysis that needs further research and tests, several different perspectives have been considered, and different themes have been addressed. The first consideration, taking into account the results achieved, is related to the cost of the HMLS system in general. It is interesting to note that, with the evolution of technology, it is possible to reach consistent and acceptable results with a lower expense for the instrument. The performances of the Stonex X120GO confirm this fact. On the other hand, it is worth mentioning that higher-level instruments of the second generation are reaching new levels of accuracy in comparison with their forerunner in the same market segment. The results achieved by the Horizon and the Orbis are going in this direction, with the Orbis providing results that are even closer to the one of the TLS reference datasets.

The acquisition strategies are still a critical part of the overall process and, as usual, the design of the survey is crucial. Nevertheless, higher-end instruments of the second generation definitely deal better with the drift error that might occur during the acquisition. For both the GeoSLAM and the Stonex in this specific test, it is clear that the longer the acquisition and the more complex the object to be surveyed, the higher the possibility that drift errors will occur. This issue is evident both in the C2C analysis and in the cross-section, and it is evident that, in some areas, the deviation with the reference TLS dataset is higher.

The results of density and feature analysis are exploiting interesting results. While the GeoSLAM and the Orbis are the ones with the upper hand in terms of the density of points (both absolute and relative), the Stonex is the one with the best results in terms of roughness. Despite the fact that it is difficult to analyse and relate the single processing variables of the different HMLS, it is interesting to highlight this information.

A further step in the evolution of this category of HMLS is probably related to the implementation of static acquisitions during the acquisition path. This solution has started to be implemented in different systems and employed in different ways. These static scans can be used both to enrich the HMLS data in terms of level of detail and to enhance the accuracy of the data collected in the form of anchor scans. The FARO Orbis possesses this feature, which is called Flash scan, and some preliminary tests have been made to analyse its functioning.

The FARO Orbis hybrid system allows for semi-static scans to be acquired along the trajectory followed by the operator during the scanning process. The acquisition time for Flash scans is 15 s, during which the operator stays in the chosen location and then resumes the mobile acquisition. The main feature of Flash scans is their ability to approach/get closer to the level of detail and accuracy typically found in traditional scans from a static sensor, as compared to mobile scanning.

**Figure 26:**
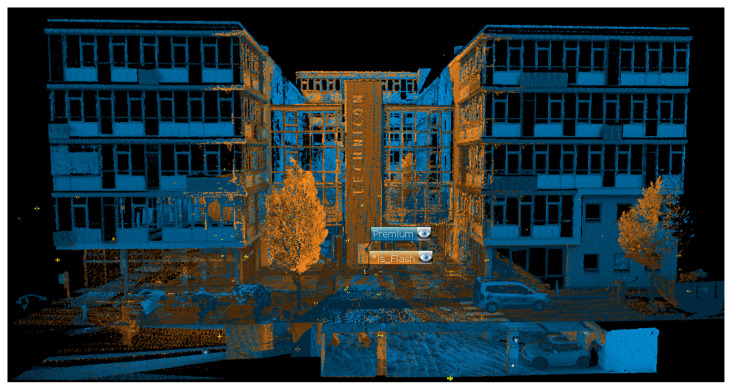
The Orbis Flash scans (orange) aligned to the static scans from Premium (blue).

Flash scans are extracted using the FARO Connect software during the processing phase of the SLAM scans and are aligned with them. So, in this study, their registration was performed through the strategy explained before for the SLAM data. In this specific case, 18 Flash scans were acquired at the same locations as the static scans performed with the Premium static sensor to compare these two datasets (Figure 26).

**Figure 27:**
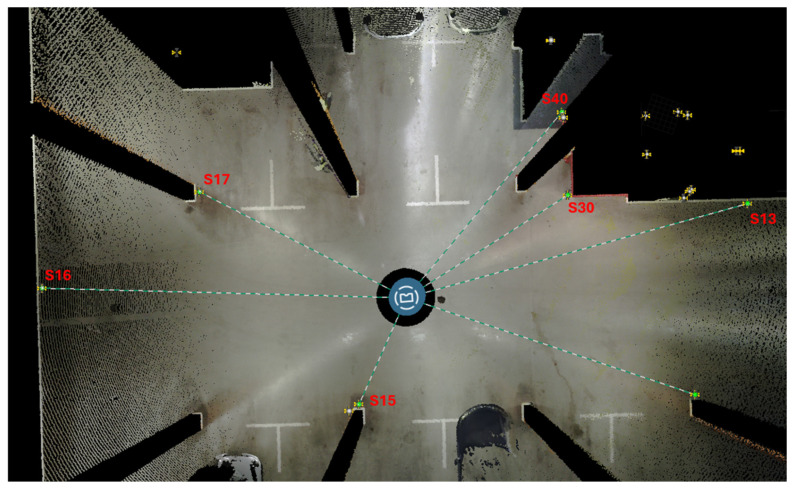
Spheres distribution around Premium scan and the Flash one.

Since the registering of FLASH scans depends on the calculation of the mobile scan based on the SLAM algorithm used, it was preferred to carry out an initial analysis by comparing a static scan, acquired by the Premium laser scanner (Res. 1/4, Qual. 3x), to the corresponding FLASH scan acquired approximately in the same position. The comparison was made using the spheres automatically extracted during the processing of both scans (Figure 27), comparing their respective deviations and the relative distances between the spheres. In the first case, deviations were highlighted by evaluating the distances between the sphere’s centre (Table 14) and in the second case, the relative distances between spheres were evaluated, showing differences between 1 and 4 mm (Table 15).

**Figure 28:**
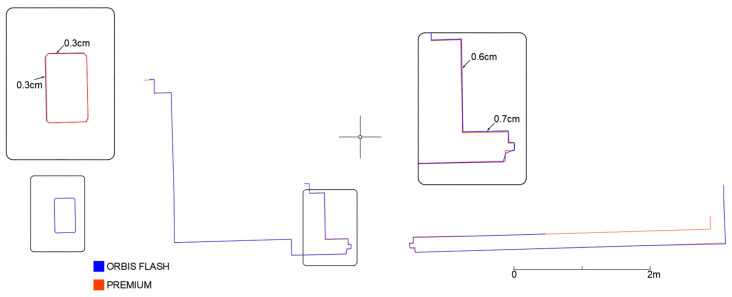
Horizontal section of a portion of the underground floor (Area 1) with flash scan.

As before, the two scans were analysed by cross-sections (Figure 28). The analysis confirms the potential of Flash technology, which, when integrated into the hybrid Orbis system, enables higher accuracy and levels of detail compared to a purely mobile approach, and produces results similar to those achieved with traditional scanning systems, but with significantly reduced acquisition times.

## 6. Conclusions and Future Works

The analysis conducted in this research allowed us to add more details and further considerations on the use of HMLS systems for the AEC sector. New-generation instruments are rapidly evolving and surpassing the performances of their forerunners. This is particularly evident in the less expensive segment of the market, where it was probably easier to implement the technology evolution at a lower cost. In the higher market segment, new instruments are finetuning their performances, adding new features, and reducing errors. All the acquisitions and processing performed in the selected test track were aimed at stressing the system performances in a complex environment, both in terms of the building complexity and in terms of different materials present (steel, glass, etc.). As stated at the beginning of the paper, the results presented in this first test are derived from acquisitions performed in a controlled environment. Further research should concentrate on more complex scenarios, also considering other environmental conditions that might affect the overall performances of the HMLS approaches (e.g., light conditions, people or construction equipment moving on the site, fog, rain, etc.) or the type of building or building site considered.

All the systems confirmed their ability to work in the range of the AEC representational scale (generally from 1:100 to 1:200) but with some differences and with the need to implement different strategies depending on the instruments used.

Finally, it needs to be reported that often, the accuracy we can reach by exploiting the capability of the first and second-generation is comparable. Nevertheless, some points need to be discussed. First of all, from the software side, the ease of use of these instruments has been implemented, allowing a wider public of operators to use these technologies. On the other hand, new functionalities like static scans are being added to the HMLS solution that are reducing the gap, at least in terms of level of detail, with traditional TLS approaches. A further step to be investigated is the possibility of generating BIM models from the HMLS data and with which level of detail and accuracy. This is an important topic that is developing, and that will need further testing and analysis.

Another important topic to be considered is related to the use of GCPs in the processing of HMLS data. This topic is adding a level of complexity to the data processing of these systems and needs ad hoc research to investigate how it affects the quality of the HMLS data both in terms of georeferencing and error mitigation. Another possible approach to be considered is the one that foresaw the use of direct georeferencing solutions using specific GNSS receivers. Finally, the possibility of applying different filtering algorithms to the data during the processing is a topic that needs to be further investigated and evaluated.

It is clear that, in several sectors, and also in the AEC and HMLS based on SLAM algorithms are emerging as a primary tool to reduce the cost and time needed to acquire the metric and geometric base supporting the creation of Digital Twins. In this sense, the platforms available on the market are not the only thing rapidly evolving, but also the research focused on the development of new algorithms and solutions. An interesting possibility can also be foreseen in the use of open-source SLAM solutions like LIO-SAM [33] or FAST-LIO [34] and FAST-LIO2 [35]. The choice of using this solution might be competitive in terms of better flexibility, lower cost, and higher customisation to the user’s needs. However, it also requires higher expertise from the operators and might be challenging for the standard users of these systems in the AEC sector. Nevertheless, this is a topic that requires further research and testing, and it might be included in future research works.

## Figures and Tables

**Table 1:** FARO Focus Premium main specifications ^1^.

Range	0.5–70/150/350 m
Max speed	Up to 2 MPts/s
3D accuracy	2 mm @ 10 m, 3.5 mm @ 25 m
Ranging error	±1 mm
Field of View	300° vertical/360° horizontal
Release date	12 April 2022

^1^ Detailed specifications can be found here: https://www.faro.com/en/Products/Hardware/Focus-Laser-Scanners (accessed on 13 April 2025).

**Table 2:** First-generation SLAM systems’ main specifications.

	GeoSLAM Horizon ^1^
Max Range	100 m
Lidar Type	Velodyne VLP
LiDAR channels	16
Points per second	300,000 points per second
Field of View	360° × 270°
Accuracy	Vertical angular resolution 2°
	Horizontal angular resolution 0.2°
Weight	1450 g + 1450 g (data logger)
Camera	Yes
RTK	No

^1^ Detailed specifications can be found here: https://geoslam.com/solutions/zeb-horizon/ (accessed on 19 February 2025).

**Table 3:** Second-generation SLAM systems’ main specifications.

	Stonex X120 GO ^1^	FARO Orbis ^2^
Max Range	120 m	120 m
Lidar Type	Hesai XT-16	-
LiDAR channels	16	32
Points per second	320,000 points per second	640,000 points per second
Field of View	360° × 270°	360° × 290°
Accuracy	6 mm	5 mm 2 mm (Flash scan)
Weight	1600 g	2100 g + 1500 (datalogger)
Camera	Yes (5 MP for each camera—3 cameras)	Yes (8 MP)
RTK	Yes	No
App (project management on-site)	Yes (for real-time acquisition)	Yes (Stream)

^1^ Detailed specifications can be found here https://www.stonex.it/project/x120go-slam-laser-scanner/ (accessed 13 April 2025) ^2^ Detailed specifications can be found here: https://www.faro.com/ (accessed on 13 April 2025).

**Table 4:** Georeferencing results of the different tested systems. FARO Focus Premium was used as the reference dataset.

Point Number	Distance Between the Couples of Points (m)		
	Horizon	Orbis	X120GO
1	0.029	0.016	0.073
2	0.040	0.086	0.087
3	0.026	0.031	0.114
4	0.050	0.056	0.044
5	0.026	0.055	0.075
6	0.027	0.081	0.087
7	0.052	0.075	0.066
8	0.057	0.016	0.029
9	0.065	0.042	0.088
10	0.074	0.013	0.127
11	0.073	0.121	0.055
12	0.069	0.016	0.111
13	0.089	0.045	0.075
14	0.111	0.066	0.092
**Mean**	**0.056**	**0.051**	**0.081**

**Table 5:** Horizontal density analysis.

Sample Area	Number of Points		
	Horizon	Orbis	X120GO
Ground floor (17 m^2^)	628.618	435.691	123.706
Underground floor (10 m^2^)	118.928	80.798	27.876
1st floor (2 m^2^)	35.184	75.944	9.380

**Table 6:** Vertical density analysis.

	Number of Points		
	Horizon	Orbis	X120GO
Ground floor (7 m^2^)	251.047	435.093	62.132
Underground floor (15 m^2^)	174.390	134.660	48.629
1st floor corridor (3 m^2^)	121.623	244.054	16.165

**Table 7:** Feature analysis. Number of neighbours on **horizontal** surfaces.

Sample Area	Number of Neighbours (r = 0.02 m); Mean/std.dev		
	Horizon	Orbis	X120GO
Ground floor (17 m^2^)	15.9/6.1	61.9/27.8	5.9/1.6
Underground floor (10 m^2^)	13.9/6.2	9.6/3.8	4.4/1.8
1st floor (2 m^2^)	61.9/60.8	35.9/17.7	9.3/2.2

**Table 8:** Feature analysis. Number of neighbours on **vertical** surfaces.

Sample Area	Number of Neighbours (r = 0.02 m); Mean/std.dev		
	Horizon	Orbis	X120GO
1st floor corridor (3 m^2^)	29.1/13.2	86.4/20.6	6.7/2
Underground floor (15 m^2^)	10.1/4.8	10.7/4.3	4.5/2
Ground floor (7 m^2^)	25.9/10.5	71.71/30.4	10.6/2.3

**Table 9:** Feature analysis. Roughness on **horizontal** surfaces.

Sample Area	Roughness (r = 0.02 m); Mean/std.dev		
	Horizon	Orbis	X120GO
Ground floor (17 m^2^)	0.0038/0.0026	0.0035/0.0025	0.0001/0.0001
Underground floor (10 m^2^)	0.0036/0.0027	0.0037/0.0029	0.0011/0.0013
1st floor (2 m^2^)	0.0036/0.0027	0.0028/0.0021	0.0001/0.0013

**Table 10:** Feature analysis. Roughness on **vertical** surfaces.

Sample Area	Roughness (r = 0.02 m); Mean/std.dev		
	Horizon	Orbis	X120GO
1st floor corridor (3 m^2^)	0.0032/0.0024	0.0029/0.0023	0.0001/0.0001
Underground floor (15 m^2^)	0.0036/0.0027	0.0036/0.0027	0.0013/0.0015
Ground floor (7 m^2^)	0.0034/0.0026	0.0037/0.0028	0.0012/0.0014

**Table 11:** C2C Analysis. Area 1 underground distribution of the distances.

	<0.02 m	<0.04 m	<0.06 m	<0.08 m
TLS-Horizon	69%	95%	96%	97.5%
TLS-Orbis	75%	98%	99%	99.5%
TLS-X120GO	83%	96%	98%	99%

**Table 12:** C2C Analysis. Area 2 ground floor.

	<0.02 m	<0.04 m	<0.06 m	<0.08 m
TLS-Horizon	79%	94%	96.5%	97.5%
TLS-Orbis	75%	93.5%	96%	97%
TLS-X120GO	44%	85%	94.5%	96%

**Table 13:** C2C Analysis. Area 3 first floor.

	<0.02 m	<0.04 m	<0.06 m	<0.08 m
TLS-Horizon	17%	79%	96%	99%
TLS-Orbis	79%	99%	99%	99%
TLS-X120GO	2%	5%	47%	99%

**Table 14:** Distances between spheres and scans positions (Premium-P and Flash-F scans), number of points detected to automatically extract the spheres in SCENE, distance between sphere centres.

Sphere	Distance (From Premium Scan Position [m])	Distance (From Orbis Scan Position [m])	N. of Points (P)	N. of Points (F)	Difference (Between P/F [m])
15	3.668	3.748	3639	2715	0.0255
30	6.036	5.952	1946	1099	0.0155
17	7.336	7.357	1246	751	0.0285
40	7.628	7.550	647	522	0.0174
13	11.144	11.086	524	318	0.0135
16	11.481	11.531	458	307	0.0452

**Table 15:** Distances between spheres.

Distance [m]	Premium	Flash
Dist S16-S17	5.81	5.809
Dist S17-S40	11.697	11.698
Dist S40-S30	2.658	2.662
Dist S30-S13	5.688	5.685
Dist S13-S15	13.767	13.771
Dist S15-S16	10.642	10.641
Dist S17-S15	8.387	8.384
Dist S17-S30	11.634	11.635
Dist S30-S15	9.301	9.304
Dist S16-S13	22.376	22.376

## Data Availability

The data presented in this study are available on request from the corresponding author due to the 3D recording of offices and spaces where R&D activities are carried out.
